# Competencies needed for public health workforce in the programs of Iran’s health transformation plan: A descriptive study based on programs, operations, and competencies chain

**DOI:** 10.1371/journal.pone.0305967

**Published:** 2024-09-23

**Authors:** Parvin Mohammadi, Nayeb Fadaei Dehcheshmeh

**Affiliations:** 1 Department of Midwifery, School of Nursing and Midwifery, Ahvaz Jundishapur University of Medical Sciences, Ahvaz, Iran; 2 Department of Public Health, Shoushtar Faculty of Medical Sciences, Shoushtar, Iran; Lorestan University of Medical Sciences, ISLAMIC REPUBLIC OF IRAN

## Abstract

**Background and purpose:**

The public health workforce faces a wide range of challenges related to people’s health. Thus, they need a combination of different professional skills and competencies to perform essential public health operations and provide services to people. The present study was conducted to determine the competencies needed for the public health workforce to implement health transformation plan programs at Ahvaz University of Medical Sciences in the capital of Khuzestan province in Iran.

**Methods:**

This research was a descriptive cross-sectional study that was conducted in 2020 among 51 managers and experts of the health deputy and faculty members of the public health school. STROBE guideline was used for the present study. The research tools were two researcher-made questionnaires based on the chain of Programs, Operations, and Competencies (POC). Additionally, the validity and reliability of the questionnaires were checked. Cronbach’s alpha coefficient was equal to 0.87 for the first questionnaire (programs-operations) and 0.85 for the second questionnaire (operations-competencies).

**Results:**

The operations of surveillance of population health and well-being, monitoring and responding to health hazards and emergencies, and disease prevention, including early detection of illness, obtained the highest mean total score (3.9 (95% CI: 3.6 to 4.2)). Respect for and adherence to ethical principles and confidentiality in any professional activity obtained the highest mean total score (4.0 (95% CI: 3.7 to 4.3)) among the needed competencies.

**Conclusion:**

To properly implement public health programs, managers must plan and take necessary measures to improve such operations as surveillance of population health and well-being, monitoring and responding to health hazards and emergencies, disease prevention (including early detection of illness), and promotion of the workforce competencies in the field of respect for and adherence to ethical principles and confidentiality in any professional activity.

## Introduction

During the last few years, there has been a lot of emphasis at the international level on measuring competencies and performance in health care [1]. To provide health services and speed up the advancement of healthcare technology, the competencies of the public health workforce have been considered to plan and achieve high-quality healthcare [2]. Competence is a combination of knowledge, abilities, skills, and values needed to perform a professional activity. In the Plan of the Association of Schools of Public Health in the Europe Region (ASPHER) for the Public Health Core Competencies, the concept of public health competencies has been defined as the intellectual or practical capacity or ability to implement defined public health operations or activities [3].

The public health workforce faces a wide range of challenges related to people’s health in the world [4]. These challenges range from demographic changes such as the obesity epidemic and the aging of the population in developed countries to the huge burden of poor health resulting from poverty and limited resources in developing countries. A competent workforce and the necessary infrastructure can play a vital and important role in dealing with these complex issues [5]. Therefore, the list of core intellectual (knowledge) and practical (skills) competencies related to public health should be continuously developed to face these challenges, and the competencies should reflect the developments and advancements resulting from scientific and technological research on the activities in the field of public health [6]. In 2008, the Public Health Agency of Canada released the “Core Competencies for Public Health in Canada,” which outline the necessary knowledge, skills, and attitudes for practicing public health. It includes 36 core competency statements in 7 categories (Public Health Sciences; Assessment and Analysis; Policy and Program Planning, Implementation, and Evaluation; Partnerships, Collaboration, and Advocacy; Diversity and Inclusiveness; Communication; and Leadership) and serves as a baseline for fulfilling core functions in the public health system [7]. The 2021 report from the Chief Public Health Officer of Canada states that public health systems focus on improving overall health, addressing health disparities, and preventing health crises [8]. 15 competencies on surveillance, outbreak detection, interview skills, investigation team, specimen testing, data analysis, hypothesis generation, study design, communication, enteric disease biology, control measures, legal authority, quality improvement, environmental health, and reporting to surveillance were developed for public health professionals by white et al. [9].

The educational system of medical sciences in Iran has been facing problems since the previous years that require specific reforms, among which the incompatibility of the provided education with the current health needs of the country can be mentioned [10].

Today, it has been demonstrated that the need to address newer requirements by managers and executives in various professional and specialized fields is increasing day by day. Successful organizations have achieved this and emphasize capacity building and promotion of professional and specialized capabilities of human resources at all levels instead of emphasizing general goals [11]. Human resources form the basis of healthcare systems and if their educational plans and programs are not appropriate to the professional needs and social conditions of the countries, they will not be able to promote health in their communities to a level that people have socially and economically a productive life [12]. A combination of different and professional skills is needed to provide primary healthcare services [13]. Thus, considering the implementation of the health transformation plan, one of the basic principles for creating the changes is the preparation of infrastructure, especially human resources, and proper planning [14]. Based on their facilities, skills, and knowledge acquired from education, human resources can play a great role in improving and increasing productivity in different stages [11].

Ghanbari et al. (2016) emphasized in their study that the provision of infrastructure, which is an important part of the prerequisites of the health transformation plan, has been neglected, especially in the field of training of specialized workforce and technology. One of the most important current problems of the transformation plan is the lack of sufficient knowledge and efficient human resources [15]. Rabiei et al. (2015) recognized the provision of experienced human resources according to the needs of executive groups as one of the solutions for revising the health transformation plan [16]. Despite these studies, the competencies required for the implementation of essential public health operations have not been addressed and this issue has received less attention. Considering that the health transformation plan has been notified and implemented in cities and their outskirts since 2015, it is necessary to identify the competencies and training required for essential public health operations according to the prospect of the health transformation plan. Therefore, to achieve this goal, using a new approach based on the chain of Programs, Operations, and Competencies (POC), we first determined the essential operations for the implementation of public health programs in this study. Then, we presented the competencies needed by the operations.

Using the results of the present study, it is possible to determine the essential operations related to public health programs, improve the competencies, and promote the training programs for public health workforces.

## Methods

This research was a descriptive cross-sectional study that was conducted in 2020, from 01/01/2020 to 31/08/2020, at Ahvaz University of Medical Sciences in the capital of Khuzestan province in Iran. The present paper was written based on STROBE guidelines (exclusive edition for cross-sectional studies) [17].

### Study population

The study population consisted of the managers and experts of the health deputy and faculty members of the public health school. As the study population was limited, sampling was not done. In addition to being willing to participate in the study, the inclusion criteria were having at least 5 years of work experience and working in the departments providing health services. The exclusion criteria were the reluctance to answer the questionnaire and not returning the questionnaire.

### Research tools

To determine the essential operations and competencies needed for the public health workforce, two researcher-made questionnaires based on POC were used. In the first step, the essential operations for the implementation of the national programs of the health transformation plan were determined using the questionnaire of programs and operations. It should be noted that the national programs of the health transformation plan include:

1. The program of providing primary health care services (modern health services) to villagers, cities with less than 20 thousand population, and nomads.
2. The program of providing primary health care services (modern health services) to marginalized populations and cities with 20 to 50 thousand population.
3. The program of providing primary health care services (modern health services) to the cities with more than 50 thousand population and metropolises.
4. The program of family physician and the referral system.
5. The program for the promotion and development of self-care.
6. Strengthening and institutionalizing inter-sectoral cooperation
7. Public health program (safe water, safe food and healthy diet, clean air)
8. The program of oral and dental health
9. The program of prevention and control of non-communicable diseases and risk factors
10. The program of population, promoting reproductive health and childbearing
11. The program of improving and modifying the community’s nutrition pattern
12. The program of promoting mental and social health and prevention and treatment of drug abuse
13. The program of prevention and control of communicable diseases
14. The program for reducing high-risk behaviors and HIV
15. The program of health in natural and man-made disasters.

The researchers have also used these 15 programs as criteria for determining public health essential operations. As the WHO European Region has studied and determined the standards of this field for many years, the standard list provided by the WHO European Region under the title of WHO Europe’s Essential Public Health Operations (EPHOs) can be used to select essential public health operations. In addition, the standard competencies determined by the Association of Schools of Public Health in the Europe Regional (ASPHER) can be used to select competencies [6].

The standard list provided by the WHO European Region for essential public health operations includes 3 domains and 10 operations:

- Intelligence (1. Surveillance of population health and well-being and 2. Monitoring and responding to health hazards and emergencies)
- Core services delivery (1. Health protection, including environmental and occupational health, food safety, and others, 2. Health promotion, including action to address social determinants and health inequity, and 3. Disease prevention, including early detection of illness)
- Enablers (1. Assuring governance for health and well-being, 2. Assuring a sufficient and competent public health workforce, 3. Assuring sustainable organizational structures and financing, 4. Advocacy, communication, and social mobilization for health, and 5. Advancing public health research to inform policy and practice for the implementation of public health programs)

The scale used to score the questionnaire was a five-point Likert scale including the options of very high, high, medium, low, and very low. Each response was assigned a point value from five to one. The participants determined the value of the essential operations based on the Likert scale and the essential operations for the implementation of each program of the transformation plan were specified.

After determining the essential public health operations, the questionnaire of operations and competencies was used in the next step and the competencies needed to perform the essential operations of the transformation plan were determined. As mentioned above, the standard competencies determined by the Association of Schools of Public Health in the Europe Regional (ASPHER) were used to select competencies, which include 5 domains and 16 competencies:

- Methods in public health (1. Understanding the definitions, models, and concepts of health, public health, philosophy of science, sociology, social psychology, and anthropology, and 2. Applying epidemiological, statistical, and qualitative methods to concrete settings, including IT handling, needs assessment, and literature search and evaluation)
- Population health and its determinants (1. Analysis of the impact of environmental and social determinants on health and disease, 2. Identification of groups with elevated risk and recognition of their needs, and 3. Performing risk assessment and management)
- Health policy, economics, organization, and management (1. Planning, implementation, management, and evaluation of public health programs, including identification of stakeholders and establishment of partnerships, 2. Performing health economic and health impact assessment, SWOT analysis, and organization analysis, and 3. having insight into own leadership style).
- Health promotion and education (1. Knowing and applying the main health promotion concepts (empowerment, holism, community development, participation, capacity building, social marketing, and health advocacy), 2. Identifying population health challenges, 3. Communicating effectively public health messages to different audiences by using modern media, 4. Planning, implementation, management, and evaluation of the strategies of health protection and communicable disease control, and 5. Environmental health management and disease prevention (primary, secondary, and tertiary))
- Ethics (1. Identifying the ethical aspects of public health interventions, strategies, and policies, 2. Ensuring the implementation of basic ethical principles in public health strategy, such as a nondiscriminatory approach, and 3. Respecting and adhering to ethical principles and confidentiality in any professional activity).

The scale used to score the questionnaire was a five-point Likert scale including the options of very high, high, medium, low, and very low. Each response was assigned a point value from five to one. The participants determined the value of the competencies based on the Likert scale and the competencies required to perform the operations selected for each program.

To examine the face and content of validity, the questionnaires were given to 10 experts and faculty members who had at least five years of work experience in sections providing health services. It should be noted that these people did not participate in the main study. The face validity of the questionnaires was evaluated with a qualitative approach in terms of the level of difficulty and the degree of inadequacy and ambiguity in the questions. Content validity was evaluated using the two factors of content validity ratio (CVR) and content validity index (CVI). The necessity and usefulness were examined by CVR and the simplicity, transparency, and relevance of each question were examined by CVI. The scores obtained were reviewed in a meeting with expert professors and the necessary corrections were made to the questionnaires. The content validity ratio (CVR) of 0.83 was obtained for the questionnaire of programs and operations and 0.86 for the questionnaire of operations and competencies. The CVI of the questionnaires was also acceptable (0.75–1.00). The reliability of the questionnaire was also evaluated in terms of internal consistency using Cronbach’s alpha coefficient. Cronbach’s alpha coefficient was equal to 0.87 for the first questionnaire (programs and operations) and 0.85 for the second questionnaire (operations and competencies).

### Data collection and analysis

After receiving permission to carry out the research, two briefing sessions were first held for the study population in the meeting hall of the health deputy and the health schools. Based on the research information sheet, necessary training and guidance regarding the objectives, benefits of the study, and how to complete the questionnaires were presented and the questionnaires were distributed. It should be noted that a personal visit to the workplace of the people who did not attend the meetings was made to justify them and distribute the questionnaires. Then, the researcher again visited the workplaces of the individuals and collected the questionnaires. Additionally, the questionnaires were anonymous and the ethical principles of the research were respected by keeping the information confidential. The data were analyzed using descriptive indices such as percentage, mean, and standard deviation.

### Ethical statement

This study was approved by the Institutional Review Board (IRB) of the Shoushtar Faculty of Medical Sciences and registered under number 98000038. Additionally, the proposal of this study was also approved by the ethics committee of the same faculty and registered under code IR.SHOUSHTAR.REC.1398.001.Main, the methods were carried out following the relevant guidelines and regulations. Written consent was obtained in each case.

## Results

73 questionnaires were distributed and the number of participants in this study was 51. The response rate was almost 70% due to the weak participation of faculty members of the health school. 15.7% of participants were men and 84.3% were women. The average age of the studied subjects was 40.0 (7.3) years. The majority of participants (43.1%) were between 30 and 39 years old. Most of them (49.0%) had a bachelor’s degree and were permanently employed (70.6%). The job title of 64.7% of the participants was expert. The mean score for their work experience was 15.0 (6.9) years and the mean score for their management experience was 2.0 (4.4) years (Table 1).

**Table 1:** Demographic characteristics of the studied subjects.

Variables	Frequency n(%)
**Gender**	
Male	8 (15.7)
female	43 (84.3)
**Age**, mean (SD)	40.0 (7.3)
**Age**	
<30	3 (5.9)
30–39	22 (43.1)
40–49	20 (39.2)
≥50	6 (11.8)
**Education**	
Associate	5 (9.8)
Bachelor	25 (49.0)
Master	18 (35.3)
Doctor	3 (5.9)
**Employment**	
permanent	36 (70.6)
temporary-to permanent	3 (5.9)
contractual	9 (17.6)
other	3 (5.9)
**Job Title**	
Expert	33 (64.7)
Director	7 (13.7)
Chairman	9 (17.6)
Manager	1 (2.0)
Faculty member	1 (2.0)
**Work experience**, mean (SD)	15.0 (6.9)
**Management experience**, mean (SD)	2.0 (4.4)

The results showed that the mean total score of the essential public health operations was 3.7 (95% CI: 3.4 to 4.0). Among essential operations, the highest mean scores were related to surveillance of population health and well-being, monitoring and responding to health hazards and emergencies, and disease prevention, including early detection of illness (3.9 (95% CI: 3.6 to 4.2)) and advancing public health research obtained the lowest mean score (3.1 (95% CI: 2.7 to 3.5)) (Table 2).

**Table 2:** Mean and confidence interval of essential operations.

Essential Operations	Mean (95% CI)
Surveillance of population health and well-being	3.9 (3.7 to 4.2)
Monitoring and responding to health hazards and emergencies	3.9 (3.5 to 4.1)
**Intelligence**	
Health protection, including environmental, occupational, food safety, and others	3.7 (3.4 to 4.0)
Health promotion, including action to address social determinants and health inequity	3.7 (3.5 to 4.0)
Disease prevention, including early detection of illness	3.9 (3.6 to 4.2)
**Core services delivery**	
Assuring governance for health	3.7 (3.4 to 4.0)
Assuring a competent public health workforce	3.8 (3.4 to 4.1)
Assuring organizational structures and financing	3.6 (3.2 to 3.9)
Information, communication, and social mobilization for health	3.4 (3.0 to 3.8)
Advancing public health research to inform policy and practice	3.1 (2.7 to 3.5)
**Enablers**	
**Total**	3.7 (3.4 to 4.0)

**Chart 1:**
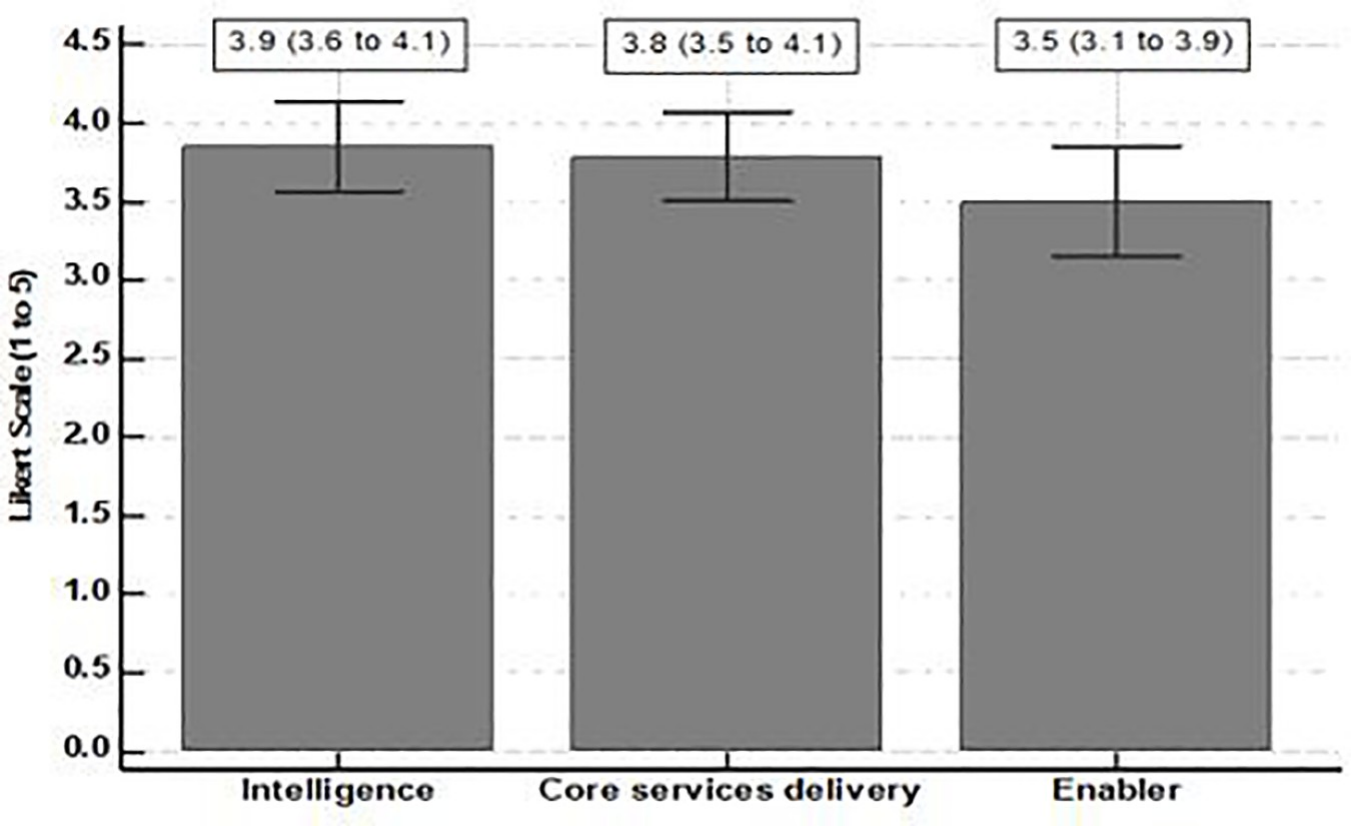
Mean and confidence interval of the domains of essential operations.

Chart 1 shows that among the domains of essential operations, intelligence was the most important one (3.9 (95% CI: 3.6 to 4.1)).

The mean total score of the competencies needed to implement public health programs was 3.7 (3.4 to 4.0). According to the results obtained, respect for and adherence to ethical principles and confidentiality in any professional activity obtained the highest mean score (4.0 (95% CI: 3.7 to 4.3)) among the required competencies. The lowest mean score (3.5 (95% CI: 3.2 to 3.9)) was related to the competencies of understanding the definitions, models, and concepts of public health, applying epidemiological, statistical, and qualitative methods to concrete settings, performing health economic and health impact assessment, SWOT analysis, and organization analysis, and having insight into own leadership style (Table 3).

**Table 3:** Mean and confidence interval of competencies.

Competencies	Mean (95% CI)
Definitions, concepts & models	3.5 (3.3 to 3.8)
Epidemiological, statistical & qualitative methods	3.5 (3.2 to 3.8)
**Methods in public health**	
Analyze the impact of environmental and social determinants on health and diseases	3.8 (3.4 to 4.1)
Identify groups with elevated risk, and recognize their needs	3.8 (3.5 to 4.1)
Risk assessment and management	3.7 (3.4 to 4.0)
**Population Health & Its Determinants**	
Plan, implement, manage, and evaluate public health programs	3.7 (3.3 to 4.0)
Health economic & organization analysis	3.5 (3.2 to 3.8)
Insight into own leadership style	3.5 (3.2 to 3.9)
**Policy; economics; organization; management**	
Know and apply main health promotion concepts	3.6 (3.3 to 3.9)
Identify population health challenges	3.7 (3.4 to 4.0)
Plan, implement, manage, and evaluate strategies for health protection and communicable disease control	3.8 (3.5 to 4.1)
Environmental health management & disease prevention (primary, secondary, tertiary)	(3.5 to 4.1)
Effectively communicate public health messages to different audiences by using modern media;	3.8 (3.5 to 4.2)
**Health promotion and education**	
Identify ethical aspects of programs	3.7 (3.5 to 4.0)
The implementation of basic ethical principles in public health strategy	3.8 (3.6 to 4.1)
Respect and adhere to ethical principles and confidentiality	4.0 (3.7 to 4.3)
**Ethics**	
**Total**	3.7 (3.4 to 4.0)

**Chart 2:**
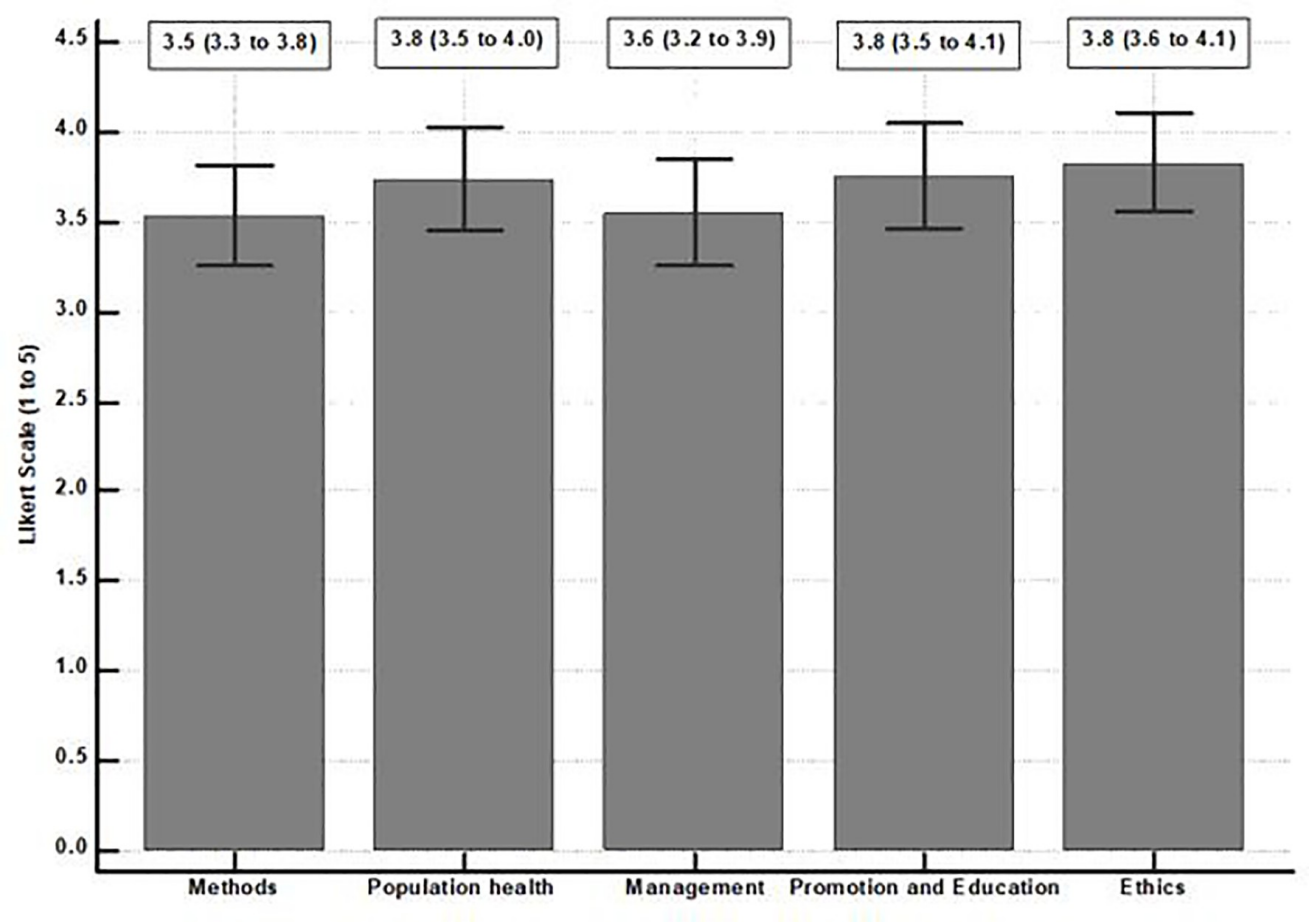
Mean and confidence interval of the domains of competencies.

As seen in Chart 2, the domains of ethics, population health and its determinants, and health promotion and education were more interesting to the respondents (3.8 (95% CI: 3.5 to 4.1)). However, competencies related to the domain of methods were less important (3.5 (95% CI: 3.3 to 3.8)).

According to Table 4, for the surveillance of population health, health protection, and health promotion, the competencies related to ethics were more important than the domains of methods and management of public health programs. Of course, in addition to ethics, population health and its determinants also had the highest mean score (3.8 (95% CI: 3.5 to 4.2) for health protection.

**Table 4:** Mean and confidence interval of the domains of competencies needed for essential operations.

Operations/Competencies	Methods	Population Health & Its Determinants	Policy; economics; organization; management	Health promotion and education	Ethics
**Surveillance**	3.6 (3.3 to 4.0)	3.9 (3.6 to 4.2)	3.6 (3.2 to 3.9)	3.8 (3.5 to 4.1)	4.0 (3.7 to 4.2)
**Monitoring**	3.7 (3.4 to 4.0)	3.9 (3.5 to 4.2)	3.6 (3.2 to 3.9)	3.9 (3.6 to 4.2)	4.0 (3.7 to 4.3)
**Health protection**	3.5 (3.2 to 3.8)	3.8 (3.5 to 4.2)	3.5 (3.2 to 3.9)	3.7 (3.4 to 4.1)	3.8 (3.6 to 4.1)
**Health promotion**	3.5 (3.2 to 3.8)	3.7 (3.4 to 4.1)	3.5 (3.1 to 3.8)	3.7 (3.4 to 4.1)	3.8 (3.5 to 4.1)
**Disease prevention**	3.7 (3.3 to 4.0)	3.7 (3.4 to 4.1)	3.5 (3.2 to 3.8)	3.7 (3.4 to 4.0)	3.7 (3.4 to 4.0)
**Governance**	3.7 (3.4 to 4.0)	3.9 (3.6 to 4.1)	3.8 (3.5 to 4.1)	3.9 (3.7 to 4.2)	4.1 (3.8 to 4.3)
**Public health workforce**	3.6 (3.3 to 3.9)	3.8 (3.5 to 4.1)	3.8 (3.5 to 4.0)	3.9 (3.7 to 4.2)	4.0 (3.7 to 4.3)
**Organizational and financing**	3.4 (3.0 to 3.7)	3.6 (3.3 to 4.0)	3.6 (3.3 to 3.9)	3.6 (3.3 to 4.0)	3.7 (3.4 to 4.0)
**Communication**	3.2 (2.8 to 3.6)	3.5 (3.2 to 3.9)	3.4 (3.1 to 3.8)	3.6 (3.3 to 4.0)	3.7 (3.3 to 4.0)
**Research**	3.4 (3.0 to 3.8)	3.4 (3.0 to 3.8)	3.2 (2.9 to 3.6)	3.4 (3.0 to 3.8)	3.4 (3.0 to 3.8)

Additionally, like the previous operations, paying attention to ethics had a significant role in monitoring and responding to health hazards and emergencies, measures related to governance, training the competent workforce, organizational structures and financing, communication, and research on public health. However, for the implementation of these operations, management (3.6 (95% CI: 3.2 to 3.9)) was less important.

For disease prevention, including early detection of illness, the lowest mean score was related to the domain of management (3.5 (95% CI: 3.1 to 3.9)) and the mean score for other domains was the same (3.7 (95% CI: 3.3 to 4.1)).

Table 5 shows that among the domains of competencies, the most required competency for intelligence is ethics (4.0 (95% CI: 3.7 to 4.3)) and management had the least importance (3.6 (95% CI: 3.3 to 3.9)). It was recognized that for the core services delivery, ethics and the population health and its determinants (3.8 (95% CI: 3.5 to 4.1)) had more priority than methods and management (3.5 (95% CI: 3.2 to 3.8)). The highest mean score for competencies in the domain of enablers was related to ethics (3.8 (95% CI: 3.5 to 4.1)) and the lowest mean score was related to methods (3.5 (95% CI: 3.2 to 3.8)).

**Table 5:** Mean and confidence interval of the domains of required competencies based on the separation of the domains of essential operations.

Operations/Competencies	Methods	Population Health & Its Determinants	Policy; economics; organization; management	Health promotion and education	Ethics
**Intelligence**	3.7 (3.4 to 4.0)	3.9 (3.6 to 4.2)	3.6 (3.3 to 3.9)	3.8 (3.6 to 4.1)	4.0 (3.7 to 4.3)
**Core services delivery**	3.5 (3.2 to 3.8)	3.8 (3.5 to 4.1)	3.5 (3.2 to 3.8)	3.7 (3.4 to 4.0)	3.8 (3.5 to 4.1)
**Enablers**	3.5 (3.2 to 3.8)	3.7 (3.4 to 4.0)	3.6 (3.3 to 3.9)	3.7 (3.4 to 4.1)	3.8 (3.5 to 4.1)

## Discussion

Determining the essential operations and competencies needed for public health workforces to implement programs of health transformation plan was the main purpose of the present study. As no study has been done on the chain of programs, operations, and competencies in Iran so far, we used this practical model to identify the essential operations for the implementation of public health programs. Then, we obtained very interesting results regarding the competencies needed for essential public health operations. In this study, we recognized that intelligence was more important than other domains of essential operations for public health programs. Surveillance of population health and well-being, monitoring and responding to health hazards and emergencies, and disease prevention, including early detection of illness, were determined as the most important essential operations while advancing public health research was less important. Ethics was more important than other domains of competencies for the implementation of essential public health operations. Respect for and adherence to ethical principles and confidentiality in any professional activity was considered the most important competency needed for the implementation of public health programs. The domains of population health and its determinants and health promotion and education were in the next degrees of importance. The competencies related to the domains of the methods and management, including understanding the definitions, models, and concepts of public health, applying epidemiological, statistical, and qualitative methods to concrete settings, performing health economic and health impact assessment, SWOT analysis, and organization analysis, and having insight into own leadership style were considered less important.

For the surveillance of population health, public health workforces must be able to identify high-risk groups and their needs, and while respecting and adhering to ethical principles, they must also observe the principle of confidentiality. Khamebini et al. found that there was a significant relationship between the technical, communication, and ethical skills of human resources for working in health and treatment centers [18]. Additionally, in the study of Abotalebi and Biglu, personal and ethical competencies were identified as the main competencies [19]. The findings of Tegene et al. showed that health professionals had a limited attitude toward patient confidentiality and suggested providing a continuous medical ethics training package for them before employment [20]. These findings are in line with the results of the present study.

The present study also showed that the workforce should be able to communicate effectively public health messages to different audiences by using modern media to monitor and respond to health hazards and emergencies. The workforce must be well trained and have the necessary skills to effectively respond to acute public health events. Communication skills are necessary for the implementation of public health interventions and community participation during crises and hazards [21]. On the other hand, the findings showed that communicating effectively public health messages to different audiences using modern media and ensuring the implementation of basic ethical principles in public health strategy play a decisive role in disease prevention, including early detection of illness. Various studies show that insufficient knowledge of nutrition and the inability to communicate it to society on the part of health workforces are the factors contributing to the failure in the prevention of cardiovascular diseases [22]. In a study in Singapore, the information gap between providers and patients was the main ethical issue when screening patients. This study showed that the harms of unnecessary tests may be greater than their benefits [23]. For public health experts to be able to provide surveillance of population health by preventing and early detection of diseases and responding timely to hazards and emergencies, they should be empowered in the field of identifying the needs of vulnerable groups, communicating health messages, and observing ethical principles through intelligence. For this purpose, their communication and ethical skills need to be strengthened.

In general, intelligence is of particular importance in public health programs. The findings of the present study showed that the public health workforce needs more competencies and capabilities in the field of ethical principles to implement this operation. The public health system has significant ethical challenges. Health professionals distrust the legitimacy, utility, and privacy of the public health system. Educational materials and ethics guidance for the programs of the public health system is limited and scattered [24].

Another essential public health operation is the delivery of core services. In this study, it was emphasized that the implementers of public health programs should have the necessary competence in the field of recognizing the determinants of population health and observing ethical principles to deliver appropriate services. Yeshineh et al. (2022) argued that compliance with healthcare ethics is an inseparable part of the current activities of health and treatment centers. The poor knowledge of healthcare ethics by health professionals leads to many unethical practices in their daily activities [25]. According to Rheinsberg et al., physicians are obliged to act in the patient’s best interests in a way that strengthens the impact of knowing their physical and mental conditions during the provision of healthcare [26]. Inevitably, most healthcare workers, especially the frontline workforce, commit unethical and unacceptable practices [27]. The results of other studies and the present study confirm that the knowledge and skills of public health workforces need to be strengthened to behave ethically when delivering services.

In this study, we recognized that in the field of enablers, it is important to pay attention to the competencies related to ethics. It is necessary to recognize the ethical dimensions of public health interventions, strategies, and policies. Additionally, it was found in the studies related to the SARS outbreak that governments, public health, and healthcare workers predominantly raised ethical issues as opposed to logistical and scientific issues [28]. Policy recommendations should be feasible in the health system and the intrusiveness of interventions should be justified by the public health benefits [23]. The results show that ensuring the implementation of basic ethical principles in public health strategy plays an important role in policymaking and program implementation and it is necessary for public health executives to be competent in this field.

It should be noted that research activities were less important among the essential operations for the public health programs in the present study. In the study by Brownson et al., it was pointed out that public health organizations need sufficient capacity (availability of resources, structures, and manpower to plan, deliver, and evaluate the preventive dose of evidence-based intervention) to move science to practice and often a set of new skills is needed to identify and implement evidence-based interventions [29]. Qari et al. emphasized the importance of the participation of public health service providers in research and the identification of practice-based research questions [30]. This is not consistent with the results of the present study. It seems that the reason for this is the difference in the approach of different countries to research. The fact that employees in public health service centers only provide services and do not actively participate in research activities can undermine the quality and effectiveness of public health programs and interventions.

In this research, less emphasis was placed on the competencies related to the management of the programs, including the implementation of health economic and organization analysis and having insight into own leadership style. Sadeghi-Ghotbabadi et al. (2013) also found that there is less emphasis on the competencies related to the management and leadership of nutrition programs and the technical skills of public health nutritionists are more important [31]. It is in line with the findings of the present study. It seems that due to the sectorial perspective, experts in any field focus more on technical skills, and management and leadership skills are less important for performing related tasks. Management and leadership skills are vital for an efficient and effective response to public health events [21]. Increasing the knowledge and strengthening the skills and abilities of public health workforces in the field of management (for example, the system analysis and leadership styles) can be effective in improving their other abilities, and not paying attention to these competencies will bring challenges to the implementation of public health programs.

Although the competencies related to the domain of methods, including understanding the definitions, models, and concepts of public health, epidemiological, statistical, and qualitative methods to concrete settings, were considered less important for essential public health operations in the present study, other studies pointed out to the basic need for these skills to have a desirable function [21]. It seems that applying and objectifying definitions and concepts of public health and epidemiological and statistical methods and models play a decisive role in the implementation of public health programs and should receive more attention.

### Limitations of the study

This research was accompanied by limitations, some of which include the low number of study samples due to the weak participation of faculty members of the health school. Considering that they have a very important role in the training of the public health workforce, their opinions and knowledge could be influential in determining the competencies needed for public health programs. In future studies, the role of university professors in improving the competencies needed for public health professionals can be addressed. Another limitation of this study was that the people participating in the study may have a bias towards the programs related to their specialization and assigned tasks, which can affect the findings. Thus, to increase the generalizability, it is suggested that in future studies, wider research should be done on the competencies needed for public health programs and the study population should be selected based on expertise and duties. Operations and competencies can be defined and studied more concretely and objectively. In addition, similar studies were not conducted in Iran and this challenge limited the use of the results of previous studies and the comparison with the findings of the present study.

## Conclusion

Based on the results of this study, operations such as surveillance of population health, monitoring and responding to health hazards and emergencies, and disease prevention, including early detection of illness, are more necessary for the implementation of public health programs of the health transformation plan. To carry out these operations, respect for and adherence to ethical principles and confidentiality in any professional activity are among the most important competencies. To implement optimally the public health programs of the health transformation plan of medical sciences universities, public health managers must plan and take necessary measures in the field of improving these operations and the competencies needed for human resources. Considering that competencies related to ethics play an important role in public health services and most of the studies on healthcare ethics have been related to clinical services, the investigation of ethical aspects of operations and competencies needed for public health workforces is felt more than ever.

## Supporting information

S1 Data Data used for analysis. (XLSX)
